# Double Reverse Traction‐Assisted Versus Traditional Freehand Closed Reduction With Hexapod External Fixator in Treating Open Tibial Shaft Fractures: A Retrospective Study

**DOI:** 10.1111/os.70239

**Published:** 2025-12-30

**Authors:** Zhiming Zhao, Yuanyuan Geng, Bowen Shi, Jian Chen, Yabin Liu, Chengkuo Cai, Guoqi Ji, Weiguo Xu

**Affiliations:** ^1^ Department of Traumatic Orthopaedics Tianjin Hospital Tianjin China; ^2^ Department of Radiology Tianjin Hospital Tianjin China

**Keywords:** closed fracture reduction, hexapod external fixator, open tibial shaft fracture, skeletal traction reduction

## Abstract

**Objective:**

Open tibial shaft fractures (OTSFs) pose significant therapeutic challenges due to high‐energy trauma, extensive soft tissue damage, and contamination risks, complicating fracture stabilization and increasing infection rates. Conventional freehand closed reduction often requires multiple attempts, exacerbating soft tissue injury and radiation exposure. To address these limitations, this study evaluates a double reverse traction‐assisted technique, hypothesizing that it could improve reduction accuracy and reduce complications in OTSFs managed with hexapod external fixators (HEFs).

**Methods:**

This retrospective cohort study analyzed the records of 55 hospitalized patients with AO/OTA type 42‐A or 42‐B OTSFs treated with HEF between March 2020 and March 2023. Double reverse traction‐assisted closed reduction was performed on 28 patients (DRTA group), while traditional freehand closed reduction was performed on 27 patients (Freehand group). We documented fracture reduction time, fluoroscopy time, external fixation time, radiographic results, electronic prescription count, and complications. Final clinical outcomes were assessed using the Association for the Study and Application of the Method of Ilizarov (ASAMI) criteria at a mean follow‐up of 15.3 months. Statistical analysis was performed using independent samples *t*‐tests or the chi‐square test.

**Results:**

DRTA group demonstrated significantly shorter fracture reduction time (12.13 ± 2.12 vs. 17.14 ± 3.43 min; *p* < 0.001) and fluoroscopy time (8.12 ± 1.78 vs. 13.75 ± 2.62; *p* < 0.001) compared to the Freehand group. External fixation time showed no significant difference (*p* > 0.05). DRTA group exhibited superior radiographic alignment, with significantly reduced residual translation and angulation on AP/lateral views (all *p* < 0.05). The electronic prescription count for postoperative correction was significantly lower in the DRTA group (0.9 ± 0.7 vs. 1.4 ± 1.0; *p* < 0.05). The complication rate was lower in the DRTA group (32.1%) than in the Freehand group (48.1%), but this difference was not statistically significant (*p* > 0.05). ASAMI scores were similar between both groups (*p* > 0.05). ASAMI bone and functional scores were similar between groups.

**Conclusion:**

In this retrospective study, both reduction techniques achieved favorable therapeutic outcomes. However, the double reverse traction‐assisted technique was associated with greater efficiency in fracture reduction, more accurate radiographic alignment, and a nonsignificant trend toward lower complications compared to traditional freehand reduction. These results indicate that the double reverse traction‐assisted technique is a feasible and promising alternative, but its definitive advantages need to be confirmed by larger, prospective, randomized controlled trials.

## Graphical abstract

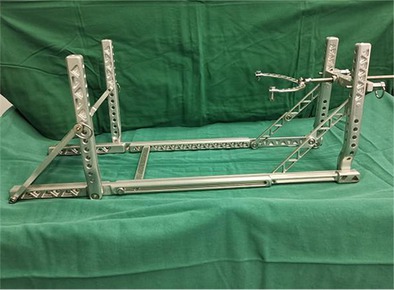

## 1 Introduction

Open tibial shaft fractures (OTSFs) represent a prevalent form of orthopedic trauma characterized by concomitant skin and soft tissue injuries, posing significant therapeutic challenges. Effective management necessitates a multidisciplinary approach addressing fracture alignment, soft tissue reconstruction, and infection control [1, 2]. External fixation systems are widely employed in OTSF management due to their minimal soft tissue requirements, reduced iatrogenic trauma via percutaneous application, and lower postoperative infection risks compared to conventional techniques [3, 4]. The hexapod external fixator (HEF), an advanced circular fixation system, enables three‐dimensional fracture correction through computer‐assisted manipulation of six adjustable struts, permitting precise spatial realignment of fractures. It can provide multidirectional and stable fixation effects and has good mechanical stability. In recent years, its application has gradually increased and has become an important tool for treating OTSFs. However, clinical adoption has shortcomings, such as high technical costs and a long learning curve [5, 6, 7, 8].

Freehand closed reduction remains a cornerstone technique in fracture management, offering advantages such as minimized soft tissue disruption, reduced infection risks, and enhanced fracture healing potential [9, 10, 11]. Based on the closed reduction of fractures, the application of HEF can combine the advantages of both techniques to create better conditions for treating OTSFs, which is conducive to promoting the union of tibial shaft fractures and reducing fracture‐related complications.

**FIGURE 1:**
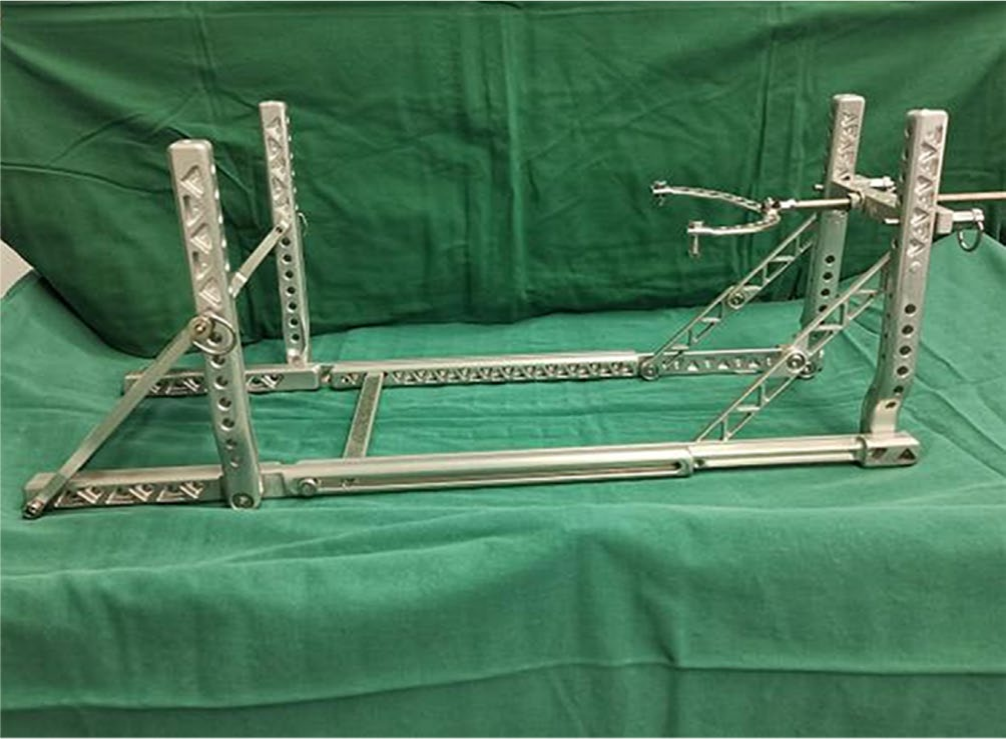
Double reverse traction‐assisted apparatus.

A critical limitation of conventional closed reduction lies in the absence of direct visual fracture site access, frequently resulting in suboptimal reduction quality due to inadequate fragment control. Iterative manipulation attempts often prolong the operative duration and exacerbate peri‐fracture vascular compromise. They also necessitate excessive fluoroscopy imaging, thereby elevating radiation exposure risks. This could potentially increase the radiation risk for both patients and medical staff [9, 12]. Therefore, improving the accuracy of closed reduction is the key to improving the treatment effect of OTSFs. Double reverse traction‐assisted technology is an emerging reduction assistance method that applies traction forces in opposite directions at both ends of the fracture to help better align the fracture ends, improving the reduction accuracy. In recent years, the application of the double reverse traction apparatus (DRTA) in lower limb fractures of patients in China has gradually increased [13, 14]. However, robust clinical evidence comparing its efficacy to conventional freehand techniques in the management of HEF‐managed OTSFs is still lacking. The purpose of this study was to (i) compare the procedural efficiency and reduction quality between double reverse traction‐assisted and traditional freehand closed reduction for OTSFs managed with HEF; and (ii) evaluate the subsequent clinical outcomes and complication profiles associated with each reduction technique. DRTA is shown in Figure 1.

## 2 Materials and Methods

### 2.1 General Information

This retrospective cohort study included 55 patients with OTSFs (classified as OTA/AO 42‐A or 42‐B) treated at the Department of Traumatic Orthopaedics, Tianjin Hospital, between March 2020 and March 2023, following predefined inclusion/exclusion criteria.

The inclusion criteria were as follows: (i) age range of 18–70 years old, (ii) Gustilo–Anderson classification II–IIIB OTSFs, (iii) HEF application with either double reverse traction‐assisted or traditional freehand closed reduction, (iv) HEF was used as the definitive fixation method, and (v) Patients were followed for a minimum of 12 months after surgery.

The exclusion criteria were as follows: (i) documented cognitive impairment or psychiatric conditions, (ii) the patient cannot tolerate long‐term use of an external fixator, and (iii) inability to comply with follow‐up protocols.

We allocated patients to the DRTA or Freehand group based on preoperative planning rather than randomization. As this was a retrospective study, the choice of reduction technique was determined by the surgical team before the operation, considering the specific characteristics of the fracture and the planned application of the HEF. To minimize operator bias, the decision was made according to a standardized protocol and was not influenced by the individual surgeon's preference or proficiency on the day of surgery. This approach ensured that the allocation was consistent and based on clinical judgment rather than arbitrary selection.

The study included 36 males and 19 females with an age range of 18–70 years. Eight patients presented with concomitant skin/soft tissue defects (12–24 cm^2^) and universal fibular fractures. One patient had a concurrent fracture of the left seventh rib, while another patient experienced a fracture of the right medial malleolus. Twenty‐eight patients were treated with double reverse traction‐assisted closed reduction, and 27 patients were treated with traditional freehand closed reduction. No significant differences were observed in the demographic characteristics between the DRTA group and Freehand group (*p* > 0.05), suggesting that they were comparable (Table 1). The study protocol was approved by the Tianjin Hospital Institutional Review Board (approval number: 2025‐015) and was conducted in accordance with the principles of the Declaration of Helsinki. Written informed consent was obtained from all participants before their inclusion.

**TABLE 1:** Demographics in the DRTA group and Freehand group.

Item	DRTA group (*n* = 28)	Freehand group (*n* = 27)	*p*
Gender			0.506
Male	20 (71.4)	16 (59.3)	
Female	8 (28.6)	11 (40.7)	
Age (year)	35.2 ± 8.2	38.4 ± 8.3	0.156
Injury mechanism			0.719
Road traffic accident	18 (64.3)	19 (70.4)	
High fall injury	6 (21.4)	3 (11.1)	
Crush injury	3 (10.7)	3 (11.1)	
Sports injury	1 (3.6)	2 (7.4)	
Side of injured			0.869
Left	12 (42.9)	10 (37.0)	
Right	16 (57.1)	17 (63.0)	
AO/OTA type of fractures			0.960
42‐A1	6 (21.4)	4 (14.8)	
42‐A2	5 (17.9)	6 (22.2)	
42‐A3	8 (28.6)	7 (26.0)	
42‐B2	5 (17.9)	6 (22.2)	
42‐B3	4 (14.2)	4 (14.8)	
Gustilo–Anderson classification			0.420
Type II	5 (17.9)	9 (33.3)	
Type IIIA	14 (50.0)	11 (40.7)	
Type IIIB	9 (32.1)	7 (26.0)	
Time from the injury to HEF installation (days)	4.9 ± 1.5	5.2 ± 1.7	0.491

Abbreviation: HEF, hexapod external fixator.

Indications for surgery: all patients required surgical intervention due to high‐energy open fractures with significant soft tissue damage (Gustilo–Anderson classification II–IIIB), which necessitated immediate stabilization to prevent infection, promote healing, and restore limb alignment. Selection criteria for HEF and reduction techniques: HEF was chosen as the definitive fixation method for its minimal soft tissue dissection and suitability for contaminated wounds. Patients were allocated to either the DRTA group or the Freehand group based on preoperative planning, aiming to compare efficiency and outcomes between these techniques. Both groups met identical anatomical (AO/OTA type 42‐A/B) and injury severity criteria, ensuring comparability.

### 2.2 Preoperative Preparation

All patients received emergent debridement, primary wound closure, and calcaneal skeletal traction in the Emergency Department before admission to the orthopedic trauma unit. Once the wound showed no signs of inflammation and either returned to or approached normal, the installation of HEF was carried out following debridement. All procedures and postoperative evaluations were performed by a single orthopedic trauma team to minimize inter‐operator variability.

### 2.3 Surgical Technique

All surgical procedures were performed by a single orthopedic trauma team, which included two senior attending surgeons with more than 10 years of experience in complex trauma management and external fixation, along with their standardized resident assistants. This approach was implemented to minimize inter‐operator variability and ensure the comparability of surgical skills between the two groups. The choice of reduction technique (double reverse traction‐assisted or traditional freehand) was based on the surgical plan developed preoperatively and was not influenced by the individual surgeon's preference or proficiency on the day of surgery.

#### 2.3.1 Fixation of DRTA Group and Freehand Group

Procedures were conducted under general or epidural anesthesia with patients positioned supine on a radiolucent operating table to facilitate intraoperative imaging.

DRTA group: With the knee flexed at 30°, the DRTA (Tianjin Pengzhi Haiger Medical Technology Co. Ltd) was positioned beneath the lower leg, followed by insertion of the pre‐assembled HEF system. A 2.0‐mm K‐wire was inserted into the calcaneus and securely connected to the traction arch of the DRTA. The traction force was applied incrementally by rotating the handle clockwise. The initial traction force was empirically set at approximately 10%–15% of the patient's body weight (e.g., ~8–12 kg for an 80 kg patient) to overcome muscle spasm and achieve initial length restoration. The final traction force was adjusted intraoperatively under fluoroscopic guidance based on the fracture morphology (e.g., greater traction for highly comminuted 42‐B3 fractures to maintain length, and less for simple 42‐A1 patterns to avoid over‐distraction). The device allows for continuous traction force application with a maximum capacity of 30 kg. Furthermore, the traction bow can be pivoted and locked at multiple angles to facilitate the correction of angular deformities in both the coronal and sagittal planes. Under real‐time fluoroscopy, the length and alignment were restored. Under the C‐arm, 2–3 2.0‐mm olive wires were threaded through the tibial and fixed on the ring, which were subsequently tensioned. One to two 6‐mm half wires were added to reinforce each ring. In addition, six quick universal adjustment rods between two sets of rings were placed in a sliding state. The reduction of tibial shaft fractures was achieved by a DRTA applying traction under the C‐arm fluoroscopy to correct the angular and displacement deformities of tibial shaft fractures.

Freehand group: Installation of the HEF was the same as that of the DRTA group. Under fluoroscopy guidance, two surgeons performed manual bidirectional traction to correct angular/translational deformities, supplemented by percutaneous reduction clamp application when necessary. After satisfactory alignment of the fracture ends, the quick universal adjustment rods were placed in a locked state.

For patients whose wounds cannot be closed, the following methods were used: (i) regional flap reconstruction, (ii) skin grafting, and (iii) a nice knot was used to suture the wound, and the wound was gradually pulled and closed slowly after surgery. Fibular fractures located > 8 cm proximal to the ankle joint line were managed nonoperatively based on established biomechanical principles. For patients with combined medial malleolus fractures, open reduction is performed, followed by fixation with 3.5‐mm hollow screws (Shandong Weigao Orthopedic Materials Co. Ltd., China), and rib fractures were treated conservatively.

#### 2.3.2 Postoperative Management

**FIGURE 2:**
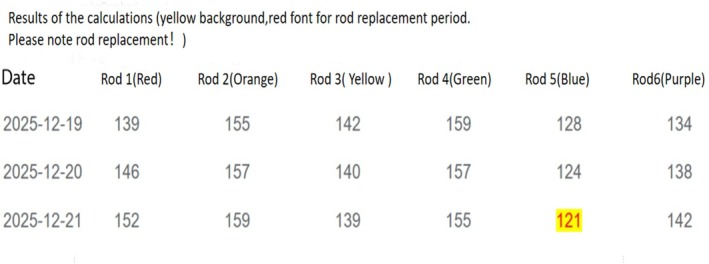
Screenshot of the CareFix Frame software interface showing the generation of an electronic prescription for strut adjustment following postoperative radiographic evaluation.

Postoperative Day 1 (POD1): Patients initiated isometric quadriceps strengthening alongside active‐assisted range‐of‐motion exercises for the knee and ankle joints. POD2: Supervised by physiotherapists, patients began partial weight‐bearing ambulation using bilateral axillary crutches and progressed to structured rehabilitation protocols. POD3: Standardized AP and lateral radiographs were obtained to evaluate fracture alignment accuracy. Residual deformities exceeding defined thresholds (angulation > 5° or translational displacement > 3 mm) were considered suboptimal and indicated for correction. For these cases, the following process was undertaken: (1) Measurement: Key parameters were measured from the postoperative radiographs, including the mounting parameters (spatial relationship of the rings to the bone), deformity parameters (magnitude and direction of the residual angulation and translation in all three planes), and fixator parameters (initial strut lengths and ring diameter). (2) Prescription generation: These parameters were input into the HEF's proprietary computer software (CareFix Frame software, Version 2.1, Shanghai CareFix Pharmaceutical Technology Co. Ltd., China). The software, based on its internal coordinate transformation algorithms, automatically generated an “electronic prescription”—a set of instructions detailing the exact length adjustments (in millimeters) required for each of the six struts to achieve the desired correction. A representative screenshot of the software interface displaying the electronic prescription is shown in Figure 2. (3) Execution: The struts were manually adjusted according to this prescription. A confirmatory radiograph was typically obtained after the adjustment to verify improved alignment. The “electronic prescription count” thus represents the number of such software‐guided correction cycles required for each patient during the entire treatment period, serving as an inverse indicator of the quality of the initial intraoperative reduction. Monthly radiographic surveillance via AP/lateral views monitored fracture union progression until external fixator removal. The decision to remove the fixator was made when the following radiographic and clinical criteria were met simultaneously: Radiographically, bridging callus had to be present across at least three cortices on both anteroposterior and lateral views, indicating robust fracture union, with “bridging” defined as a continuous, uninterrupted structure connecting the main fracture fragments. Clinically, the patient had to be able to bear full weight on the injured limb without pain at the fracture site and exhibit no tenderness upon direct palpation. The final decision was confirmed by consensus of at least two senior surgeons, based on the most recent follow‐up radiographic and clinical examination.

#### 2.3.3 Evaluation

The fracture reduction time, fluoroscopy time, external fixation time, radiological results, electronic prescription count, and complications were documented between the two groups. At the final follow‐up, the ASAMI score was employed to assess bone healing and functional recovery [15]. The ASAMI criteria, a comprehensive system for assessing bone healing, categorizes outcomes into four distinct tiers based on several factors. These include the extent of bone regeneration, the occurrence of infection, the correction of angular deformities, and any discrepancy in lower limb length. The final prognosis is then assigned a rating of either excellent, good, fair, or poor. For lower limb function recovery, ASAMI employs a six‐tier scoring system that takes into account various aspects: the return of joint mobility, the level of pain experienced, the presence or absence of intermittent claudication, the condition of local tissue health, the degree of knee and ankle joint contractures, and any reduction in the range of motion for both the knee and ankle joints. This assessment culminates in a prognostic classification that is also divided into excellent, good, fair, or poor.

#### 2.3.4 Statistical Analysis

The statistical analysis was conducted utilizing SPSS version 21.0 (IBM Corp, USA). Continuous variables (e.g., fracture reduction time, fluoroscopy time, external fixation time, follow‐up time, translation and angulation on AP/lateral views, electronic prescription count) were analyzed by independent‐samples *t*‐tests and are expressed as mean ± standard deviation (mean ± SD). Categorical variables (e.g., gender, injury mechanism, side of injury, AO/OTA type, Gustilo–Anderson classification, types of complications, ASAMI bone and functional scores) were analyzed by the chi‐square test or Fisher's exact test, and are reported as counts (percentages). A *p* value of less than 0.05 was considered to indicate statistical significance.

## 3 Results

### 3.1 Patient Follow‐Up

The mean follow‐up time was similar between the DRTA group (15.3 ± 0.6 months) and the Freehand group (15.4 ± 0.8 months) (*p* > 0.05), with no significant difference observed. The overall mean follow‐up time was 15.3 months (range: 12–18 months). All patients completed 12–18 months of postoperative follow‐up.

### 3.2 Procedural Efficiency and Radiographic Outcomes

DRTA group demonstrated significantly shorter fracture reduction times compared to the Freehand group (12.13 ± 2.12 vs. 17.14 ± 3.43 min; *p* < 0.001). The fluoroscopy time was markedly lower in the DRTA group than in the Freehand group (8.12 ± 1.78 vs. 13.75 ± 2.62; *p* < 0.001). The external fixation time was (6.04 ± 0.87 months) for the DRTA group and (6.45 ± 1.23 months) for the Freehand group (*p* > 0.05). DRTA group exhibited superior radiographic alignment, with significantly reduced residual translation and angulation on AP/lateral views compared to the Freehand group (all *p* < 0.05). The electronic prescription count was (0.9 ± 0.7) for the DRTA group and (1.4 ± 1.0) for the Freehand group (*p <* 0.05) (Table 2).

**TABLE 2:** Clinical outcomes in DRTA group and Freehand group.

Item	DRTA group (*n* = 28)	Freehand group (*n* = 27)	*p*
Fracture reduction time (min)	12.13 ± 2.12	17.14 ± 3.43	< 0.001
Fluoroscopy time	8.12 ± 1.78	13.75 ± 2.62	< 0.001
External fixation time (months)	6.04 ± 0.87	6.45 ± 1.23	0.161
Follow‐up time (months)	15.3 ± 0.6	15.4 ± 0.8	0.603
Radiological results			
Translation in AP view (mm)	1.8 ± 0.6	2.4 ± 0.8	0.002
Translation in lateral view (mm)	2.1 ± 0.9	2.7 ± 1.1	0.032
Angulation in AP view (°)	2.9 ± 1.6	3.9 ± 1.8	0.034
Angulation in lateral view (°)	3.1 ± 1.4	4.1 ± 1.7	0.021
Electronic prescription count	0.9 ± 0.7	1.4 ± 1.0	0.037

Abbreviation: AP, anteroposterior.

### 3.3 Complications

No cases of vascular or nerve damage, deep infection of wounds or wire tracts, or delayed union were observed in either group. In the DRTA group, 10 patients experienced superficial pin tract infections after surgery, which were controlled after oral antibiotics. Within 24 h after surgery, two patients showed clear and pale‐yellow exudate at the site of the calcaneal traction wire insertion, which was treated with a dressing change. After 4 days, the exudate disappeared and did not progress to deep infection. In the Freehand group, 11 patients experienced superficial pin tract infections after surgery, which were controlled after oral antibiotics. Two patients with open fractures had significant wound exudation after surgery; after the dressing change, the wounds healed smoothly. Two patients with nonunion of the fracture site achieved radiographic consolidation subsequent to autologous iliac crest bone grafting. Ultimately, all 55 patients (100%) achieved fracture union within the follow‐up period. The complication rate was higher in the Freehand group (48.1%) than in the DRTA group (32.1%) (Table 3).

**TABLE 3:** Complications of the two groups.

Item	DRTA group (%)	Freehand group (%)
Superficial pin tract infection	10 (35.7%)	11 (40.7%)
Deep infection	0 (0%)	0 (0%)
Wire tract exudation	2 (7.1%)	0 (0%)
Wound exudation	0 (0%)	2 (7.4%)
Delayed union	0 (0%)	0 (0%)
Nonunion	0 (0%)	2 (7.4%)
Total patients affected	9	13
Complications	32.1% (9/28)	48.1% (13/27)

### 3.4 Final Clinical and Functional Assessment

At the final follow‐up, no significant differences in the ASAMI scores were observed between the two groups (*p* > 0.05) (Table 4).

**TABLE 4:** ASAMI scores for DRTA group and Freehand group.

Variable	Excellent	Good	Fair	Poor	Failure	*p*
Bone scores						0.322
DRTA group	25	3	0	0	0	
Freehand group	23	2	2	0	0	
Functional scores						0.913
DRTA group	24	3	1	0	0	
Freehand group	24	2	1	0	0	

**FIGURE 3:**
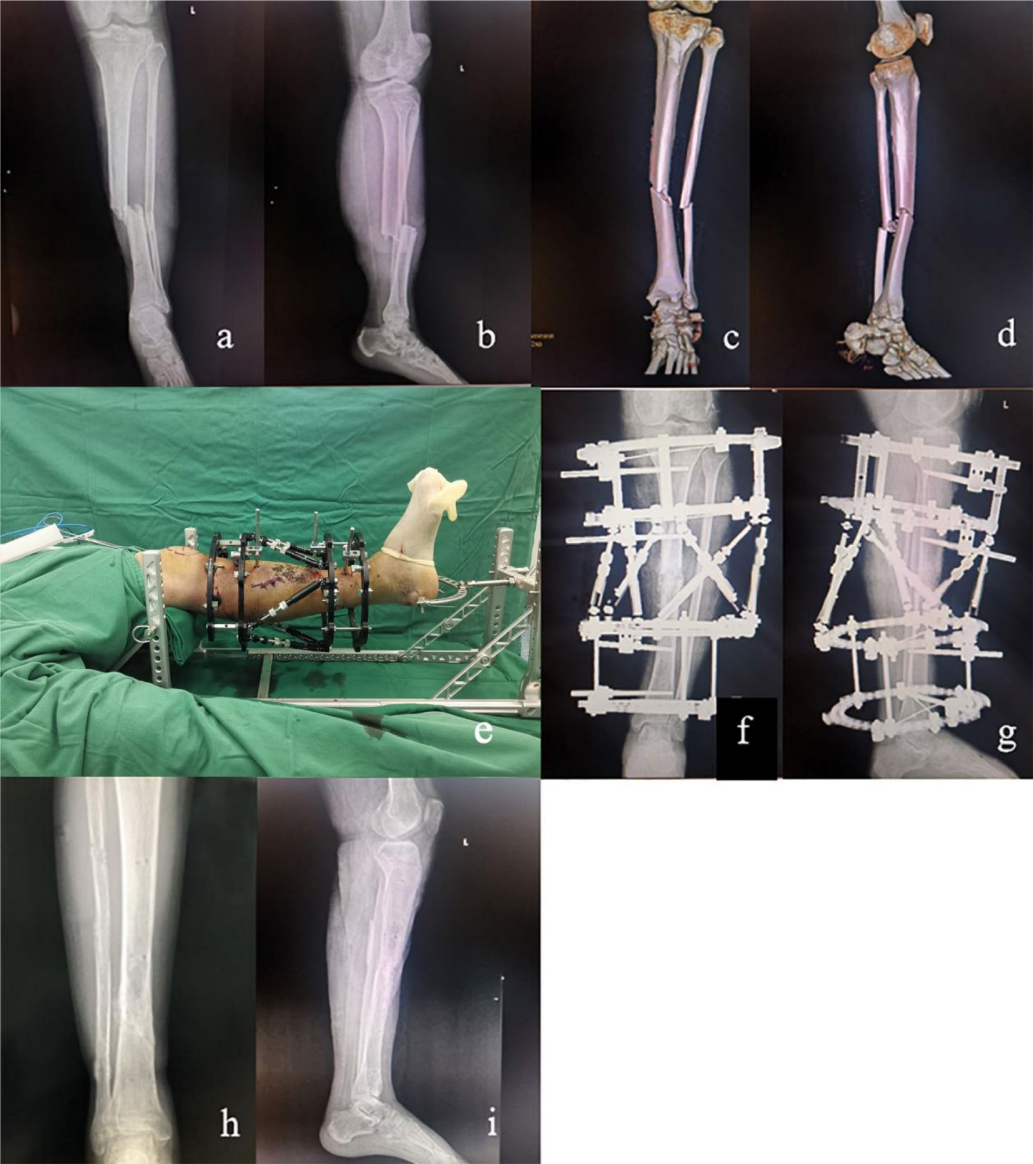
A 38‐year‐old male with Gustilo–Anderson classification IIIA open tibial shaft fracture (OTA/AO 42‐A2, simple oblique pattern) treated with double reverse traction‐assisted closed reduction and HEF. (a, b) Preoperative AP and lateral radiographs demonstrating a displaced oblique fracture with minimal comminution. (c, d) Preoperative 3D‐CT confirmed the simple oblique pattern without significant fragment separation. (e) Intraoperative photograph demonstrating the application of DRTA combined with HEF. (f, g) Immediate postoperative radiographs showing anatomical reduction with near‐perfect apposition of fracture fragments. (h, i) Final follow‐up radiographs at 6 months postoperatively (after HEF removal) showing solid bony union with excellent alignment.

**FIGURE 4:**
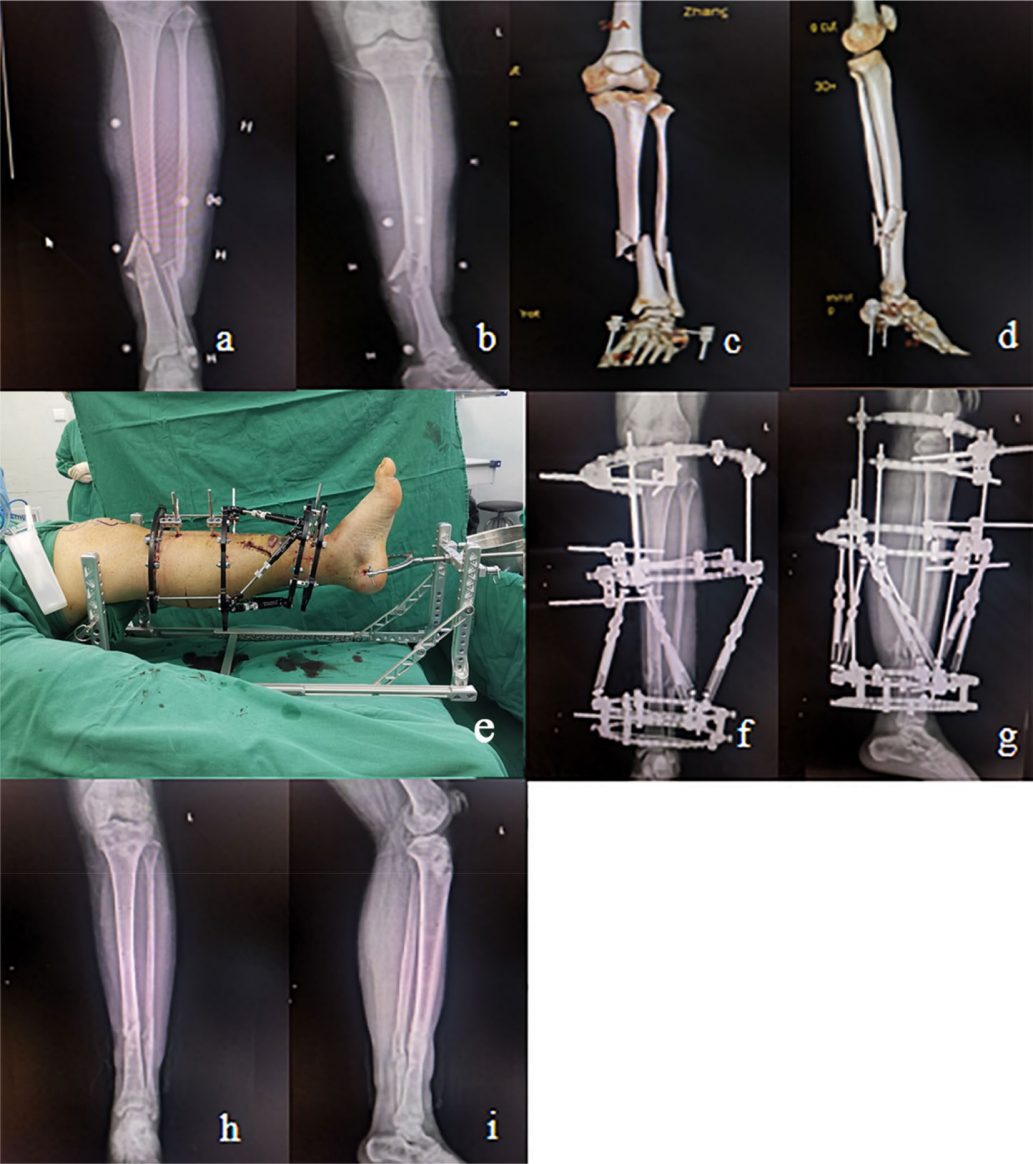
A 45‐year‐old male with Gustilo–Anderson classification IIIB open tibial shaft fracture (OTA/AO 42‐B3, highly comminuted) treated with double reverse traction‐assisted closed reduction and HEF. (a, b) Preoperative AP and lateral radiographs showed significant displacement, shortening, and comminution. (c, d) Preoperative 3D‐CT reconstructions further delineate the spiral‐oblique fracture pattern with butterfly fragments. (e) Intraoperative setup identical to Figure 3. (f, g) Postoperative AP and lateral radiographs at 1 week showing satisfactory reduction with restoration of length, alignment, and minimal residual displacement. (h, i) Final follow‐up radiographs 1 week after HEF removal (6.5 months postoperatively) demonstrating complete fracture union with good cortical continuity and alignment.

In Figures 3 and 4, two typical OTSFs are shown, which employ double reverse traction‐assisted closed reduction combined with the application of a HEF.

## 4 Discussion

### 4.1 Procedural Efficiency and Reduction Quality

Traditional freehand closed reduction is a method of fracture reduction that involves traction, folding, and other techniques. Continuous traction is required during the reduction process. When the alignment of the fracture is poor, readjustment often results in new abnormal alignment of the fracture, making fracture reduction more difficult [9]. While the DRTA for the reduction of tibial shaft fractures is capable of providing continuous traction force, the traction bow on the fixator can be adjusted according to the deformity of the fracture site to achieve a good reduction of the fracture [16]. According to the results of this study, the DRTA group demonstrated a significantly shorter fracture reduction time compared to the Freehand group (12.13 ± 2.12 vs. 17.14 ± 3.43 min, respectively; *p* < 0.001). The fluoroscopy time was markedly lower in the DRTA group than in the Freehand group (8.12 ± 1.78 vs. 13.75 ± 2.62; *p* < 0.001). DRTA group exhibited superior radiographic alignment, with significantly reduced residual translation and angulation on AP/lateral views compared to the Freehand group (all *p* < 0.05). The electronic prescription count was (0.9 ± 0.7) for the DRTA group and (1.4 ± 1.0) for the Freehand group (*p* < 0.05). In our cohort, the use of DRTA in conjunction with HEF was associated with shorter surgical and fluoroscopy times, improved radiographic outcomes, and a reduced need for postoperative adjustments to correct residual deformities. These results suggest that this combined approach could be an efficient and effective method for managing OTSFs. Shortening the surgical time means increasing patient safety, reducing the fluoroscopy time is reducing radiation damage to patients and medical staff, decreasing the electronic prescriptions count used to adjust residual deformities, reflecting enhanced initial reduction precision, and improving the quality of fracture reduction can reduce complications such as joint pain. In this study, no significant differences in the ASAMI scores were observed between the two groups.

### 4.2 Clinical Outcomes and Complications

The study compares the complication rate between the DRTA group and the Freehand group, both using the HEF for OTSFs. The overall complication rate was 32.1% for the DRTA group and 48.1% for the Freehand group. Superficial pin tract infection was the most common complication in both groups. In our clinical approach, we manage pin tract infections by administering oral antibiotics or through dressing changes. Surgical procedures are performed using low‐speed drills to minimize the risk of thermal damage. Postoperative care includes wrapping the wire tract with gauze to prevent skin movement over the olive wire. No cases of deep infection or delayed union were reported in either group. Notably, the two nonunion cases in the Freehand group (7.4%) likely resulted from periosteal devitalization during repeated reduction maneuvers, subsequently achieving union after iliac crest bone grafting. This may be associated with repeated reduction attempts and the resultant disruption of blood supply around the fracture site in the Freehand group. Consequently, the double reverse traction‐assisted reduction suggests a potential advantage in reducing the risk of nonunion. However, the findings necessitate validation in larger studies to ascertain the clinical significance and long‐term outcomes of each reduction technique.

### 4.3 Advantages and Technical Considerations of the Double Reverse Traction‐Assisted Technique

The double reverse traction‐assisted closed reduction with HEF has the following advantages in treating OTSFs: (i) Rotating the handle clockwise to apply a sustainable traction force parallel to the long axis of the tibia helps to restore the length and alignment of the tibia. Pulling in different directions with the traction arch is beneficial to correct the angular deformity of the tibial shaft fractures, thereby reducing the problem of inaccurate reduction caused by blind closure and improving the success rate of reduction. (ii) Reducing the traction and damage to surrounding soft tissues during the freehand reduction process is beneficial for protecting the integrity of soft tissues, avoiding secondary damage to soft tissues, and reducing the risk of postoperative complications [14]. (iii) This method offers superior protection for the blood supply at the fracture end, creating an advantageous biological environment promoting fracture healing [17, 18]. (iv) The combination of HEF and double reverse traction‐assisted technology can adapt to different types of OTSFs and provide an effective solution for fracture reduction. (v) The shorter surgical time also effectively reduces the risk of surgical infection. (vi) The DRTA is used for central bilateral traction, which evenly applies force to bones and soft tissues in all aspects, and can better restore the lower limb alignment, achieving more effective traction [19]. (vii) After being pulled by the DRTA, it is beneficial for the installation of the HEF and can provide sufficient operating space for the surgeon.

The following points should be noted when using a double reverse traction‐assisted fixator with HEF to treat OTSFs: (i) The application of traction force should be moderate to avoid excessive traction leading to fracture end separation or soft tissue damage [18, 20]. (ii) During the closed reduction process, the C‐arm should be used to monitor the reduction situation and ensure satisfactory alignment of the fracture end. (iii) When installing the HEF, ensure that the positions of the half wire and olive wire are correct to avoid damaging blood vessels and nerves. The half wire should pass through the contralateral cortex to ensure stability. (iv) If necessary, use point‐type reduction forceps to assist in the reduction and stabilize the fracture end. (v) Before installing the DRTA, insert the HEF into the lower leg first, paying attention to the installation sequence.

### 4.4 Comparison With Existing Literature

When contextualized within prior research, our findings affirm and clarify the role of traction‐assisted reduction. The enhanced procedural efficiency observed here mirrors outcomes from studies utilizing similar devices in femoral and tibial plateau fractures [16, 17, 18], confirming a consistent benefit in reducing operative time and radiation exposure. This validates the technique's general utility in improving intraoperative control. However, regarding clinical outcomes, a key divergence emerges. Unlike Yan et al. [17], who reported a significant reduction in major complications for intertrochanteric fractures, our study demonstrated only a nonsignificant trend. This discrepancy likely stems from fundamental differences in injury severity; our cohort consisted exclusively of high‐energy open fractures with greater inherent soft tissue compromise and infection risk, factors that may dominate the postoperative course and obscure the more subtle impact of the reduction method itself. Furthermore, the fact that superior initial radiographic alignment in our traction group did not translate into significantly better final ASAMI scores highlights the compensatory power of the hexapod fixator, which can effectively correct residual deformities postoperatively [7]. Therefore, the principal advantage of the double reverse traction technique may not be in altering the final healing benchmark but in achieving a more efficient, precise, and potentially less traumatic initial reduction.

### 4.5 Study Limitations and Future Perspectives

This study has several important limitations that warrant consideration. First, the retrospective, single‐center design and relatively small sample size (55 patients) may introduce selection bias and limit statistical power for detecting differences in less frequent outcomes. Although the sample was sufficient to evaluate primary efficiency endpoints such as reduction time and fluoroscopy time, it constrained robust analysis of uncommon complications—for instance, the occurrence of two nonunion cases exclusively in the Freehand group may be due to chance. In addition, all procedures were performed by a dedicated, experienced surgical team at a single institution, which may affect the generalizability of the findings to other settings with varying expertise or resources.

Second, the study is limited by its follow‐up duration and the scope of outcome assessment. The 12‐ to 18‐month follow‐up period, while adequate for evaluating fracture union, is insufficient to assess long‐term sequelae such as posttraumatic arthritis. More importantly, the absence of comprehensive functional and patient‐reported outcome measures (PROMs) represents a significant constraint. While radiographic union and the ASAMI score provide objective assessment of early healing, we lack objective functional metrics (e.g., knee and ankle range of motion) and validated PROMs such as the Visual Analogue Scale (VAS) for pain or the Hospital for Special Surgery (HSS) knee score. Consequently, the impact of the reduction technique on functional recovery, chronic pain, and patients' return to daily activities remains unquantified.

Third, the technique depends on a specialized commercial traction device, which requires substantial capital investment and may represent an adoption barrier, particularly in resource‐limited settings. This underscores the need for a formal cost‐effectiveness analysis.

In light of these limitations, the current study should be regarded as proof‐of‐concept, demonstrating the feasibility and potential benefits of the double reverse traction‐assisted technique within a specific clinical context. The promising trends observed here warrant validation through larger, prospective, multicenter randomized controlled trials. Future studies should include over 100 patients to improve statistical power, incorporate longer follow‐up (ideally 2–3 years or more), functional assessments, and patient‐centered outcomes, and involve diverse institutions to evaluate reproducibility and generalizability. Such research would provide higher‐level evidence regarding the technique's efficacy and broader applicability.

## 5 Conclusion

In this retrospective study, both reduction techniques achieved favorable therapeutic outcomes. However, the double reverse traction‐assisted technique was associated with greater efficiency in fracture reduction, more accurate radiographic alignment, and a nonsignificant trend toward lower complications compared to traditional freehand reduction. These results indicate that the double reverse traction‐assisted technique is a feasible and promising alternative, but its definitive advantages need to be confirmed by larger, prospective, randomized controlled trials.

## Author Contributions

**Zhiming Zhao:** writing – original draft, investigation. **Yuanyuan Geng**, **Bowen Shi**, and **Jian Chen:** formal analysis, data curation. **Yabin Liu**, **Chengkuo Cai**, and **Guoqi Ji:** data interpretation and analysis. **Weiguo Xu:** writing – review and editing, supervision. All authors read and approved the final manuscript.

## Funding

This study was supported by Tianjin Natural Science Foundation (24JCYBJC01440).

## Ethics Statement

The present study was approved by the Ethics Committee of Tianjin Hospital (approval number 2025‐015), and all patients provided written consent for their data to be used in this study.

## Conflicts of Interest

The authors declare no conflicts of interest.

## Data Availability

The data that support the findings of this study are available on request from the corresponding author. The data are not publicly available due to privacy or ethical restrictions.
